# Supplementary material for: Caffeine Intake among Undergraduate Students: Sex Differences, Sources, Motivations, and Associations with Smoking Status and Self-Reported Sleep Quality
Source: Nutrients. 2022 Apr 16;14(8):1661. doi: 10.3390/nu14081661 (PMC9029267; doi:10.3390/nu14081661)
Supplement: Supplementary file 1 [file nutrients-14-01661-s001.zip › nutrients-1634343-supplementary.pdf]

Table S1. Multivariate regression analysis for daily caffeine intake (mg·day^{-1}) in men.

Variable	B	β	95%CI	t	*p* value	R^2	Adjusted R^2	R^2 change
Smoking	100.363	0.227	48.051, 152.674	3.777	<0.001*	0.118	0.115	0.118
Alcohol	72.098	0.203	33.582, 110.614	3.685	<0.001*	0.155	0.149	0.037
Age	13.692	0.183	5.752, 21.632	3.395	0.001*	0.194	0.185	0.039
Cannabis	93.472	0.149	20.777, 166.167	2.531	0.012*	0.211	0.199	0.016
Fruit/Vegetables	12.394	0.112	0.440, 17.686	2.069	0.039*	0.223	0.209	0.012

Model: p <0.001 (ANOVA). B: regression coefficient; β: standardized beta coefficient. The positive coefficients for smoking, alcohol and cannabis indicate higher values for caffeine intake in consumers of these substances than in non-consumers. * $p < 0.05$ indicates significant predictors or R^2 changes. n = 278.

Table S2. Multivariate regression analysis for daily caffeine intake (mg·day^{-1}) in women.

Variable	B	β	95%CI	t	*p* value	R^2	Adjusted R^2	R^2 change
Smoking	79.404	0.153	38.041, 120.767	3.540	<0.001*	0.034	0.032	0.034
Fruit/Vegetables	13.975	0.166	7.515, 20.434	3.770	<0.001*	0.064	0.061	0.030
Alcohol	50.690	0.116	16.747, 84.632	4.249	0.003*	0.078	0.073	0.014
Cannabis	80.771	0.081	1.930, 159.612	2.933	0.045*	0.084	0.078	0.006
Age	1.786	0.021	−4.965, 8.537	2.012	0.604			

Model: p <0.001 (ANOVA). B: regression coefficient; β: standardized beta coefficient. The positive coefficient for smoking indicates higher values for caffeine intake in smokers than in non-smokers. * $p < 0.05$ indicates significant predictors or R^2 changes. n = 608.

Table S3. Logistic regression analysis for coffee and instant coffee intake

	Coffee			Instant coffee		
**Variable**	**OR adjusted**	**95%CI**	***p* value**	**OR adjusted**	**95%CI**	***p* value**
**Sex** (Reference men)	1.379	1.019, 1.866	0.037*	1.773	1.113, 2.826	0.016*
**Age**	1.087	1.015, 1.164	0.017*	0.946	0.858, 1.044	0.273
**Smoking** (Reference non-smokers)	2.671	1.673, 4.264	<0.001*	1.368	0.779, 2.403	0.275
**Cannabis consumption** (Reference non-consumers)	1.444	0.656, 3.178	0.361	1.242	0.481, 3.207	0.654
**Alcohol consumption** (Reference non-consumers)	2.496	1.778, 3.503	<0.001*	0.842	0.529, 1.341	0.469
**Daily fruit and vegetable servings**	1.137	1.061, 1.219	<0.001*	1.009	0.920, 1.108	0.844

*$p < 0.05$ indicates significant odds ratios (OR).

Table S4. Logistic regression for tea/mate and chocolate intake

	Tea/Mate			Chocolate		
**Variable**	**OR adjusted**	**95%CI**	***p* value**	**OR adjusted**	**95%CI**	***p* value**
**Sex** (Reference men)	2.689	1.906, 3.793	<0.001*	1.252	0.938, 1.671	0.127
**Age**	1.041	0.971, 1.116	0.261	0.976	0.915, 1.041	0.462
**Smoking** (Reference non-smokers)	0.819	0.523, 1.282	0.382	0.755	0.503, 1.134	0.175
**Cannabis consumption** (Reference non-consumers)	2.045	0.984, 4.251	0.055	1.508	0.750, 3.032	0.249
**Alcohol consumption** (Reference non-consumers)	1.083	0.761, 1.540	0.659	1.223	0.888, 1.686	0.218
**Daily fruit and vegetable servings**	1.166	1.088, 1.249	<0.001*	1.000	0.938, 1.067	0.993

* $p < 0.05$ indicates significant odds ratios (OR).

Table S5. Logistic regression for cola and energy drinks intake

	Cola drinks			Energy drinks		
**Variable**	**OR adjusted**	**95%CI**	***p* value**	**OR adjusted**	**95%CI**	***p* value**
**Sex** (Reference men)	1.030	0.748, 1.417	0.858	0.378	0.243, 0.588	<0.001*
**Age**	1.064	0.993, 1.141	0.080	0.999	0.900, 1.109	0.992
**Smoking** (Reference non-smokers)	2.103	1.389, 3.186	<0.001*	1.993	1.129, 3.519	0.017*
**Cannabis consumption** (Reference non-consumers)	0.490	0.228, 1.053	0.067	2.076	0.914, 4.715	0.081
**Alcohol consumption** (Reference non-consumers)	2.116	1.427, 3.137	<0.001*	1.208	0.688, 2.122	0.510
**Daily fruit and vegetable servings**	0.961	0.895, 1.033	0.283	0.969	0.868, 1.082	0.576

* $p < 0.05$ indicates significant odds ratios (OR).

Table S6. Multivariate regression analysis for subjective sleep quality in men

**Variable**	**B**	**β**	**95%CI**	**t**	***p* value**	**R^2**	**Adjusted R^2**	**R^2 change**
Caffeine	0.003	0.137	0.000, 0.006	2.304	0.022*	0.035	0.028	0.019
Age	−0.027	−0.016	−0.228, 0.174	−0.262	0.794			
Fruit/Vegetables	−0.248	−0.137	−0.460, −0.037	−2.310	0.022*	0.016	0.013	0.016
Smoking	0.809	0.082	−0.521, 2.140	1.198	0.232			
Cannabis	−1.168	−0.083	−2.992, 0.655	−1.261	0.295			

Regression model: p=0.007 (ANOVA). B: regression coefficient; β: standardized beta coefficient. Caffeine: caffeine intake (mg/day); Fruit/Vegetables: servings of daily fruit and vegetables intake. The positive coefficient for sex indicates higher values of MOS Sleep in women than in men. * $p < 0.05$ indicates significant predictors or R^2 changes. n = 278.

Table S7. Multivariate regression analysis for subjective sleep quality in women

Variable	B	β	95%CI	t	*p* value	R^2	Adjusted R^2	R^2 change
Caffeine	0.005	0.215	0.003, 0.006	5.338	<0.001*	0.039	0.038	0.039
Age	−0.095	−0.050	−0.244, 0.050	−1.252	0.211			
Fruit/Vegetables	−0.180	−0.099	−0.324, −0.036	−2.452	0.014*	0.049	0.046	0.006
Smoking	0.055	0.005	−0.872, 0.982	0.117	0.907			
Cannabis	−0.639	−0.029	−2.390, 1.112	−0.717	0.474			

Regression model: $p<0.001$ (ANOVA). B: regression coefficient; β: standardized beta coefficient. Caffeine: caffeine intake (mg/day); Fruit/Vegetables: servings of daily fruit and vegetables intake. The positive coefficient for sex indicates higher values of MOS Sleep in women than in men. * $p < 0.05$ indicates significant predictors or R^2 changes. n = 806.

Table S8. Results of the Caffeine Motives Questionnaire (%)

CMQ	All (*n*=807)	Men (*n*=243)	Women (*n*=564)	*p* value (Cohen's d)
CMQ Global (%)	19.29 ± 10.88	18.43 ± 10.80	19.64 ± 10.90	0.262 (0.112)
CMQ Factor 1-Cognitive enhancement (%)	38.79 ± 25.74	37.11 ± 24.73	39.45 ± 26.13	0.363 (−0.091)
CMQ Factor 2-Negative affect relief (%)	8.81 ± 17.54	7.98 ± 16.75	9.14 ± 17.85	0.508 (−0.066)
CMQ Factor 3-Reinforcing effects (%)	15.19 ± 10.86	14.98 ± 11.16	15.28 ± 10.74	0.784 (−0.027)
CMQ Factor 4-Weight control (%)	3.13 ± 8.75	1.84 ± 6.48	3.65 ± 9.46	0.016* (−0.206)

CMQ: Caffeine Motives Questionnaire. *$p < 0.05$ Indicates significant differences between men and women, as determined by Student's t-test for unpaired data. Results are expressed as mean ± SD of the percentage of the score range. Cohen's d value is provided as a measure of effect size.

Table S9. Motivations for caffeine consumption

CMQ (Score range)	All (n=807)	Men (n=243)	Women (n=564)	p value (Cohen's d)
To feel more alert	2.27 ± 1.38	2.04 ± 1.25	2.37 ± 1.41	0.011* (−0.244)
To combat a headache	1.28 ± 0.77	1.20 ± 0.63	1.31 ± 0.82	0.102 (0.146)
To help me concentrate	2.53 ± 1.42	2.49 ± 1.38	2.54 ± 1.44	0.675 (−0.042)
Because I like the taste of caffeinated beverages	3.32 ± 1.41	3.40 ± 1.37	3.29 ± 1.42	0.433 (0.078)
To help deal with stress in my daily life	1.53 ± 0.97	1.51 ± 0.99	1.54 ± 0.96	0.758 (−0.031)
To help deal with anxiety	1.29 ± 0.76	1.23 ± 0.63	1.32 ± 0.81	0.191 (−0.117)
To help deal with depression	1.24 ± 0.75	1.22 ± 0.72	1.24 ± 0.76	0.789 (−0.027)
To combat drowsiness	2.75 ± 1.60	2.72 ± 1.62	2.80 ± 1.60	0.278 (−0.109)
Because it is convenient to drink caffeinated beverages	1.11 ± 0.46	1.20 ± 0.64	1.07 ± 0.35	0.028* (0.279)
To help me focus my attention	2.51 ± 1.46	2.42 ± 1.38	2.55 ± 1.48	0.394 (−0.085)
Because I like the ''jolt'' of energy rush that I feel	2.02 ± 1.22	2.11 ± 1.31	1.99 ± 1.32	0.369 (0.090)
To help me relax or calm down	1.42 ± 0.87	1.51 ± 1.02	1.38 ± 0.81	0.177 (0.150)
To stay awake	3.23 ± 1.46	3.24 ± 1.44	3.32 ± 1.48	0.939 (0.008)
As a social past time	1.96 ± 1.16	1.89 ± 1.15	1.99 ± 1.16	0.381 (−0.088)
Because it is an ingredient in my diet pills	1.02 ± 0.21	1.03 ± 0.21	1.02 ± 0.21	0.673 (0.042)
Because I crave caffeine	1.27 ± 0.74	1.26 ± 0.74	1.28 ± 0.74	0.848 (−0.019)
As a reward to myself for completing a task	1.23 ± 0.61	1.13 ± 0.43	1.26 ± 0.66	0.008* (−0.224)
Seeing others ingest caffeine makes me crave it	1.14 ± 0.50	1.11 ± 0.45	1.15 ± 0.52	0.507 (−0.066)
Because it is a powerful diuretic	1.17 ± 0.61	1.09 ± 0.44	1.20 ± 0.67	0.022* (−0.194)
Because it puts me in a better mood	1.73 ± 1.13	1.68 ± 1.04	1.76 ± 1.16	0.494 (−0.068)
To help lose or control my weight	1.18 ± 0.62	1.11 ± 0.49	1.21 ± 0.67	0.053 (−0.170)

Values are the mean ± SD. *$p < 0.05$ Indicates significant differences between men and women, as determined by Student's t-test for unpaired data. Cohen's d value is provided as a measure of effect size.
